# Enhanced Single Image Super Resolution Method Using Lightweight Multi-Scale Channel Dense Network

**DOI:** 10.3390/s21103351

**Published:** 2021-05-12

**Authors:** Yooho Lee, Dongsan Jun, Byung-Gyu Kim, Hunjoo Lee

**Affiliations:** 1 Department of Convergence IT Engineering, Kyungnam University, Changwon 51767, Korea; yhlee@kyungnam-ispl.kr; 2 Department of IT Engineering, Sookmyung Women’s University, Seoul 04310, Korea; bg.kim@sookmyung.ac.kr; 3 Intelligent Convergence Research Laboratory, Electronics and Telecommunications Research Institute (ETRI), Daejeon 34129, Korea; hjoo@etri.re.kr

**Keywords:** deep learning, super resolution, convolutional neural network, lightweight neural network

## Abstract

Super resolution (SR) enables to generate a high-resolution (HR) image from one or more low-resolution (LR) images. Since a variety of CNN models have been recently studied in the areas of computer vision, these approaches have been combined with SR in order to provide higher image restoration. In this paper, we propose a lightweight CNN-based SR method, named multi-scale channel dense network (MCDN). In order to design the proposed network, we extracted the training images from the DIVerse 2K (DIV2K) dataset and investigated the trade-off between the SR accuracy and the network complexity. The experimental results show that the proposed method can significantly reduce the network complexity, such as the number of network parameters and total memory capacity, while maintaining slightly better or similar perceptual quality compared to the previous methods.

## 1. Introduction

**Figure 1:**
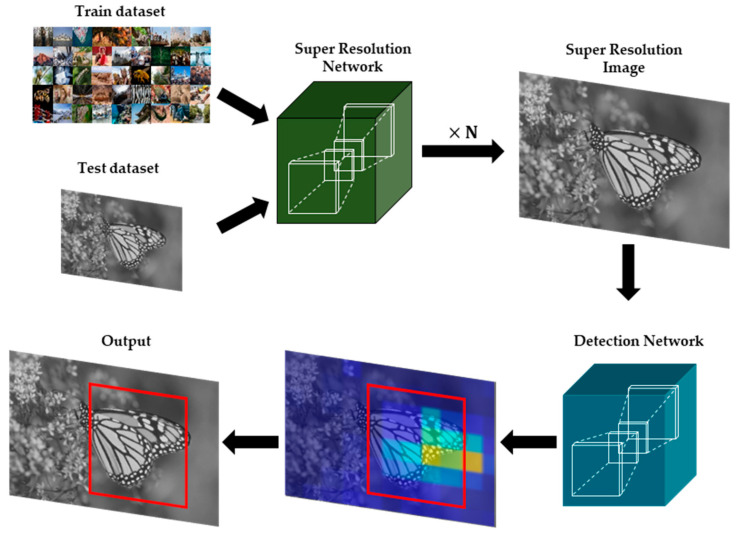
Example of CNN-based SR applications in the area of object detection.

Real-time object detection techniques have been applied to a variety of computer vision areas [1,2], such as object classification or object segmentation. Since it is mainly operated on the constrained environments, input images obtained from those environments can be deteriorated by camera noises or compression artifacts [3,4,5]. In particular, it is hard to detect objects from the images with low quality. Super resolution (SR) method aims at recovering a high-resolution (HR) image from a low-resolution (LR) image. It is primarily deployed on the various image enhancement areas, such as the preprocessing for object detection [6] of Figure 1, medical images [7,8], satellite images [9], and surveillance images [10]. In general, most SR methods can be categorized into single-image SR (SISR) [11] and multi-image SR (MISR). Deep neural network (DNN) based SR algorithms have been developed with various neural networks such as convolutional neural network (CNN), recurrent neural network (RNN), long short-term memory (LSTM), and generative adversarial network (GAN). Recently, convolutional neural network (CNN) [12] based SISR approaches can provide powerful visual enhancement in terms of peak signal-to-noise ratio (PSNR) [13] and structural similarity index measure (SSIM) [14]. 

SR was initially studied pixel-wise interpolation algorithms, such as bilinear and bicubic interpolations. Although these approaches can provide fast and straightforward implementations, it had limitations in improving SR accuracy to represent complex textures in the generated HR image. As various CNN models have been recently studied in computer vision areas, these CNN models have been applied to SISR to surpass the conventional pixel-wise interpolation methods. In order to achieve higher SR performance, several deeper and denser network architectures have been combined with the CNN-based SR networks.

**Figure 2:**
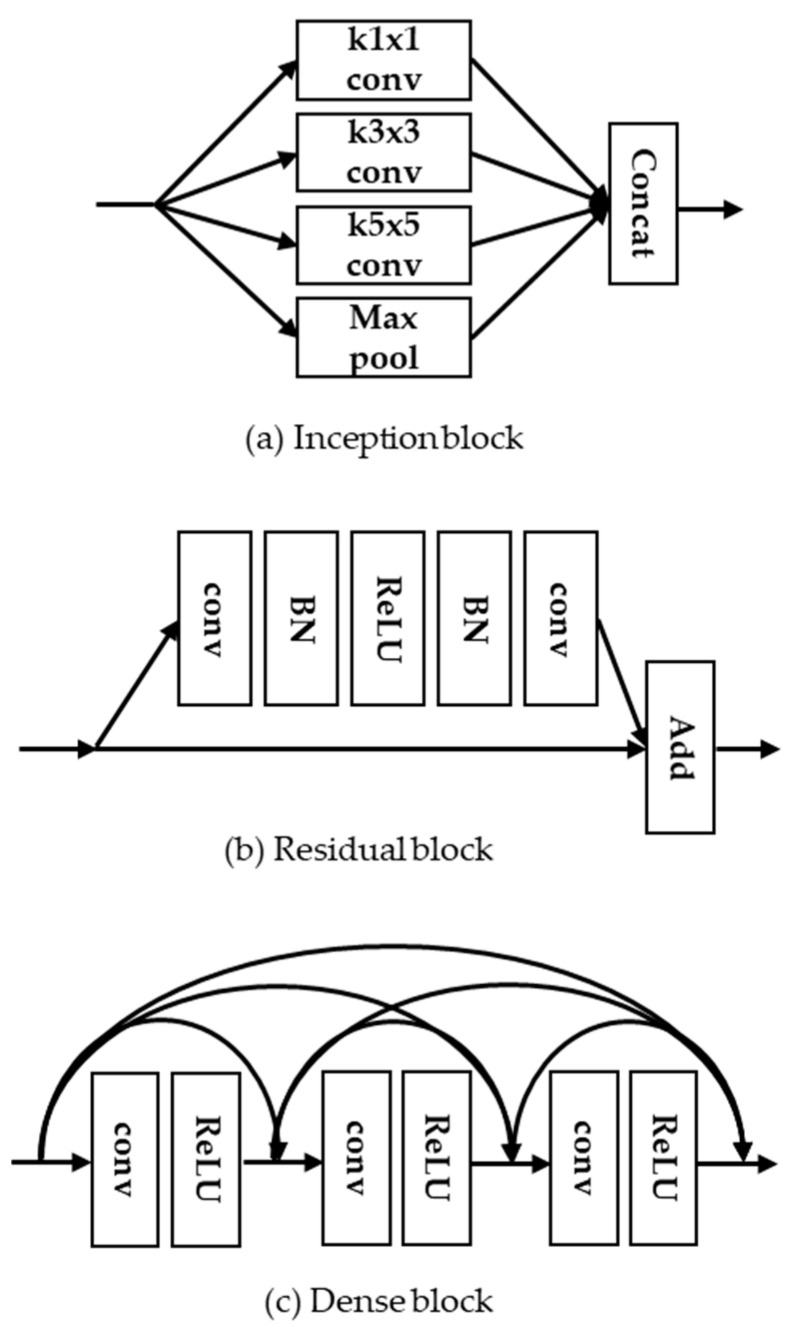
Examples of CNN-based network blocks. (**a**) Inception block; (**b**) residual block; and (**c**) dense block.

As shown in Figure 2, the inception block [15] was designed to obtain the sparse feature maps by adjusting the different kernel sizes. He et al. [16] proposed a ResNet using the residual block, which learns residual features with skip connections. It should be noted that CNN models with the residual block can support high-speed training and avoid the gradient vanishing effects. In addition, Huang et al. [17] proposed densely connected convolutional networks (DenseNet) with the concept of dense block that combines hierarchical feature maps along the convolution layers for the purpose of richer feature representations. As the feature maps of the previous convolution layer are concatenated with those of the current convolution layer within a dense block, it requires more memory capacity to store massive feature maps and network parameters. In this paper, we propose a lightweight CNN-based SR model to reduce the memory capacity as well as the network parameters. The main contributions of this paper are summarized as follows:

- We propose multi-scale channel dense block (MCDB) to design the CNN based lightweight SR network structure.
- Through a variety of ablation works, the proposed network architectures are optimized in terms of the optimal number of the dense blocks and the dense layers.
- Finally, we investigate the trade-off between the network complexity and the SR performance on publicly available test datasets compared to the previous method.

The remainder of this paper is organized as follows. In Section 2, we briefly overview the previous studies related to CNN-based SISR methods. In Section 3, we describe the proposed network framework. Finally, experimental results and conclusions are given in Section 4 and Section 5, respectively.

## 2. Related Works

In general, CNN based SR models have shown improved interpolation performances compared to the previous pixel-wise interpolation methods. Dong et al. [18] proposed a super resolution convolutional neural network (SRCNN), which consists of three convolution layers and trains an end-to-end mapping from a bicubic interpolated LR image to a HR image. After the advent of SRCNN, Dong et al. [19] proposed another fast super-resolution convolutional neural network (FSRCNN), which conducts multiple deconvolution processes at the end of the network so that this model can utilize smaller filter sizes and more convolution layers before the upscaling stage. In addition, it achieved a speedup of more than 40 times with even better quality. Shi et al. [20] proposed an efficient sub-pixel convolutional neural network (ESPCN) to train more accurate upsampling filters, which was firstly deployed in the real-time SR applications. Note that both FSRCNN and ESPCN were designed to assign deconvolution layers for upsampling at the end of the network for reducing the network complexity. Kim et al. [21] designed a very deep convolutional network (VDSR) that is composed of 20 convolution layers with a global skip connection. This method verified that contexts over large image regions are efficiently exploited by cascading small filters in a deeper network structure. SRResNet [22] was designed with multiple residual blocks and a generative adversarial network (GAN) [23] to enhance the detail of textures by using perceptual loss function. Tong et al. [24] proposed a super-resolution using dense skip connections (SRDenseNet), which consists of 8 dense blocks, and each dense block contains eight dense layers. As the feature maps of the previous convolution layer are concatenated with those of the current convolution layer within a dense block, it requires heavy memory capacity to store the network parameters and temporally generated feature maps between convolution layers. Residual dense network (RDN) [25] is composed of multiple residual dense blocks, and each RDN includes a skip connection within a dense block for the pursuit of more stable network training. As both network parameters and memory capacity are increased in the proportion of the number of dense blocks, Ahn et al. [26] proposed a cascading residual network (CARN) to reduce the network complexity. The CARN architecture was designed to add multiple cascading connections starting from each intermediate convolution layer to the others for the efficient flow of feature maps and gradients. Lim et al. [27] proposed an enhanced deep residual network for SR (EDSR), which consists of 32 residual blocks, and each residual block contains two convolution layers. Especially, EDSR removed the batch normalization process in the residual block for the speedup of network training.

Although aforementioned methods have demonstrated better SR performance, they tend to be more complicated network architectures with respect to the enormous network parameters, excessive convolution operations, and high memory usages. In order to reduce the network complexity, several researches have been studied about more lightweight SR models [28,29]. Li et al. [30] proposed multi-scale residual network (MSRN) using two bypass networks with different kernel sizes. In this way, the feature maps between bypass networks can be shared with each other so that image features are extracted at different kernel sizes. Compared to that of EDSR, MSRN reduced the number of parameters up to one-seventh, the SR performance was also substantially decreased, especially generating four times scaled SR images. Recently, Kim et al. [31] proposed a lightweight SR method (SR-ILLNN) that has 2 input layers consisting of the low-resolution image and the interpolated image. In this paper, we propose a lightweight SR model, named multi-scale channel dense network (MCDN) to provide better SR performance while reducing the network complexity significantly compared to previous methods. 

## 3. Proposed Method

### 3.1. Overall Architecture of MCDN

**Figure 3:**
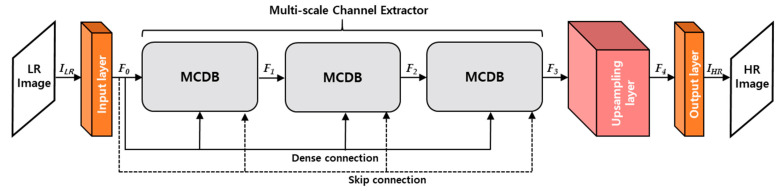
Overall architecture of the proposed MCDN.

The proposed network aims at generating a HR image whose size is 4N×4M where *N* and *M* indicate the width and height of input image, respectively. In this paper, we notate both feature maps and kernels as [W×H×C] where W×H and C are the spatially 2-dimenstional (2D) size and the number of channels, respectively. As depicted in Figure 3, MCDN is composed of 4 parts, which are input layer, multi-scale channel extractor, upsampling layer, and output layer, respectively. Particularly, the multi-scale channel extractor consists of three multi-scale channel dense blocks (MCDBs) with a skip and dense connection per a MCDB. In general, the convolution operation ($H_{i})$ of i*-th* layer calculates the feature maps ($F_{i})$ from the previous feature maps ($F_{i-1}$) as in Equation (1):

(1) $$F_{i}=H_{i}\left(F_{i-1}\right),\;where\;\;H_{i}\left(F_{i-1}\right)=\sigma \left(\;W_{i}\;⊗\;F_{i-1}+B_{i}\right),$$

where $\;F_{i-1}$, $W_{i}$, $B_{i}$, σ, and ‘⊗’ denote as the previous feature maps, kernel weights, biases, an activation function, and a weighted sum between the previous feature maps and kernel’s weights, respectively. For all convolution layers, we set the same kernel size to 3 × 3 and use zero padding to maintain the resolution of output feature maps. In Figure 3, $F_{0}$ is computed from the convolution operation of input layer ($I_{LR})$ by using Equation (2).

(2) $$F_{0}=H_{LR}\left(I_{LR}\right)=\sigma \left(\;W_{LR}\;⊗\;I_{LR}+B_{LR}\right).\;\;$$

After performing the convolution operation of input layer, $F_{0}$ is fed into the multi-scale channel extractor. The output of the multi-scale channel extractor $\left(F_{3}\right)$ is calculated by cascading MCDB operations as in Equation (3):

(3) $$F_{3}=H_{3}^{MCDB}\left(F_{2}\right)=\;H_{3}^{MCDB}\left(H_{2}^{MCDB}\left(F_{1}\right)\right)\;=\;H_{3}^{MCDB}\left(H_{2}^{MCDB}\left(H_{1}^{MCDB}\left(F_{0}\right)\right)\right),$$

where $H_{i}^{MCDB}\left(\cdot \right)$ denotes convolution operation of the i-th MCDB. Finally, an output HR image ($I_{HR}$) is generated through the convolution operations of the upsampling layer and the output layer. In the upsampling layer, we used 2 deconvolution layers with the 2 × 2 kernel size to expand the resolution by 4 times.

**Figure 4:**
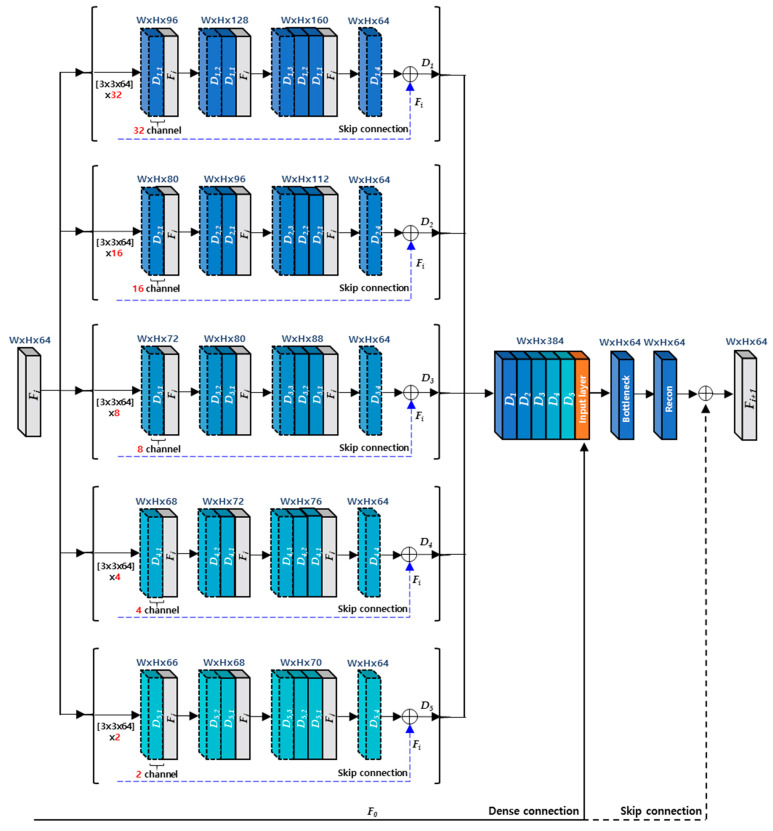
The architecture of a MCDB.

Figure 4 shows the detailed architecture about a MCDB. A MCDB has 5 dense blocks with the different channel size, and each dense block contains 4 dense layers. In order to describe the procedures of MCDB, we denote the k-th dense layer of j-th dense block as a $D_{j,k}$ in this paper. For the input feature maps ($F_{i}$), j-th dense block generates output feature maps $D_{j}$ as in Equation (4), which combine the feature maps ($D_{j,4}$) with a skip connection ($F_{i}$).

(4) $$D_{j}=D_{j,4}+F_{i},\;where\;\;D_{j,4}=H_{j,4}\left(\sigma \left(W_{j,4}⊗\;\left[D_{j,3},\;\;D_{j,2},\;\;D_{j,1},\;F_{i}\right]\right)+B_{j,4}\right).$$

After concatenating the output feature maps from all dense blocks, they are fed into a bottleneck layer in order to reduce the number of channel of the output feature maps. It means that the bottleneck layer has a role of decreasing the number of kernel weights as well as compressing the number of feature maps. The output of a MCDB is finally produced by the reconstruction layer with a global skip connection ($F_{0}$) as shown in Figure 4.

### 3.2. MCDN Training

In order to train the proposed network, we set hyper parameters as presented in Table 1. We defined L1 loss [32] as the loss function and update the network parameters, such as kernel weights and biases by using Adam optimizer [33]. The number of mini-batch size, the number of epochs, and the learning rate were set to s 128, 50, and 10^−3^ to 10^−5^, respectively. Among the various activation functions [34,35,36], parametric ReLU was used as the activation functions in our network.

## 4. Experimental Results

**Figure 5:**
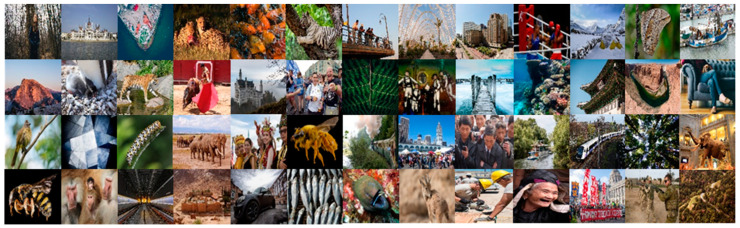
Training dataset (DIV2K [37]).

**Figure 6:**
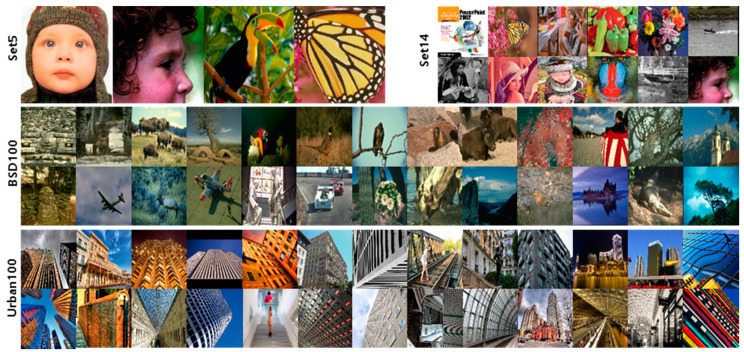
Test datasets (Set5 [38], Set14 [39], BSD100 [40], and Urban100 [41]).

As shown in Figure 5, we used DIV2K dataset [37] at the training stage. It has 2K (1920 × 1080) spatial resolution and consists of 800 images. All training images with RGB are converted into YUV color format and extracted only Y components with the patch size of 100 × 100 without overlap. In order to obtain input LR images, the patches are further down-sampled to 25 × 25 by bicubic interpolation. In order to evaluate the proposed method, we used Set5 [38], Set14 [39], BSD100 [40], and Urban100 [41] of Figure 6 as the test datasets, which are commonly used in most SR studies [42,43,44]. In addition, Set5 was also used as a validation dataset.

All experiments were conducted on an Intel Xeon Skylake (8cores@2.59GHz) having 128GB RAM and two NVIDIA Tesla V100 GPUs under the experimental environments of Table 2. For the performance comparison of the proposed MCDN, we set bicubic interpolation method as an anchor and SRCNN [18], EDSR [27], MSRN [30] and SR-ILLNN [31] are used as the comparison methods in terms of SR accuracy and network complexity.

### 4.1. Performance Measurements

In terms of network complexity, we compared the proposed MCDN with SRCNN [18], EDSR [27], MSRN [30] and SR-ILLNN [31], respectively. Table 3 shows the number of network parameters and total memory size (MB). As shown in Table 3, MCDN reduces the number of parameters and the total memory size by as low as 1.2% and 17.4% compared to EDSR, respectively. Additionally MCDN marginally reduces the total memory size by as low as 92.2% and 80.5%, respectively, compared to MSRN and SR-ILLNN with lightweight network structures. Note that MCDN was able to reduce the number of parameters significantly because the parameters used in a MCDB are identically applied to other MCDBs. 

In terms of SR accuracy, Table 4 and Table 5 show the results of PSNR and SSIM, respectively. While the proposed MCDN can significantly reduce the network complexity compared to EDSR, it has slightly high or similar PSNR performance on most test datasets. On the other hand, MCDN can achieve the improved PSNR gains as high as 0.21dB and 0.16dB on average compared to MSRN and SR-ILLNN, respectively. 

**Figure 7:**
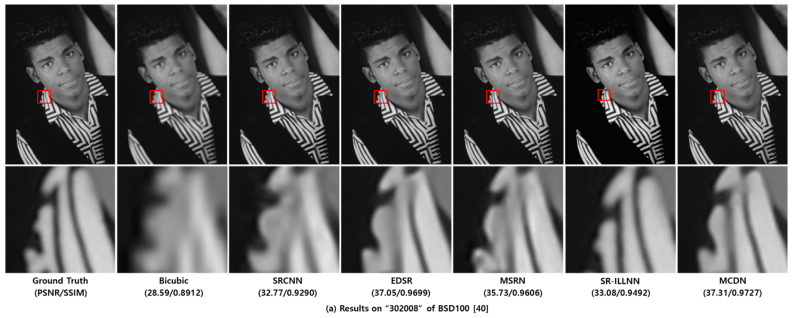

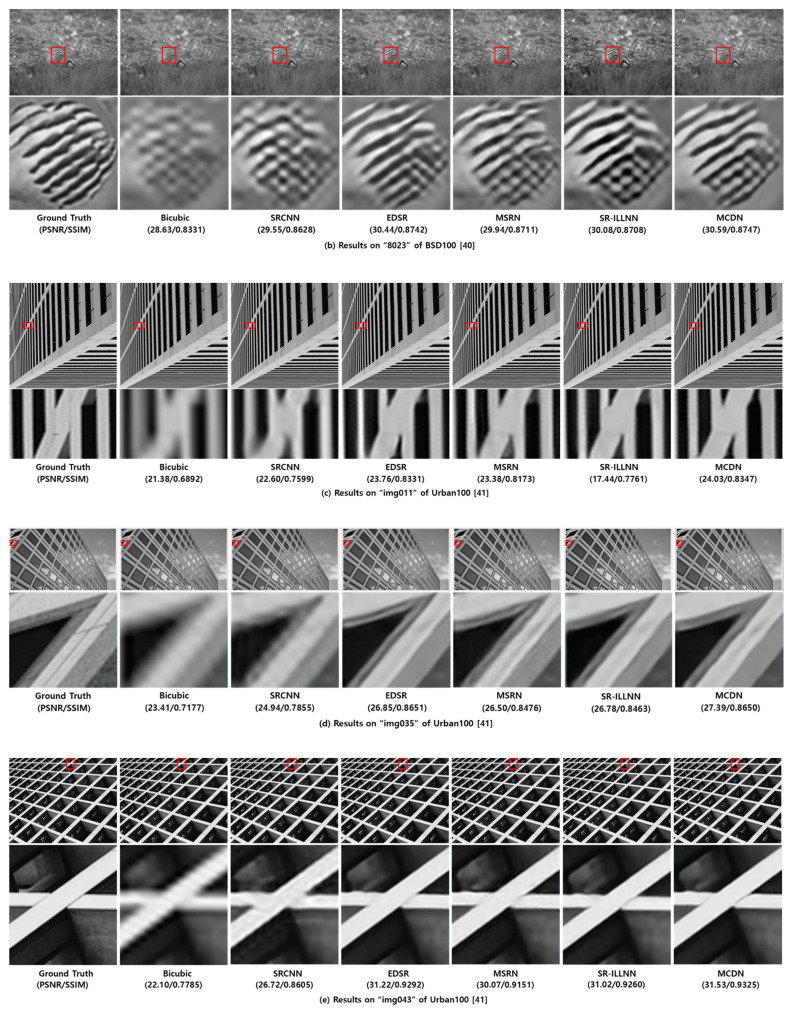

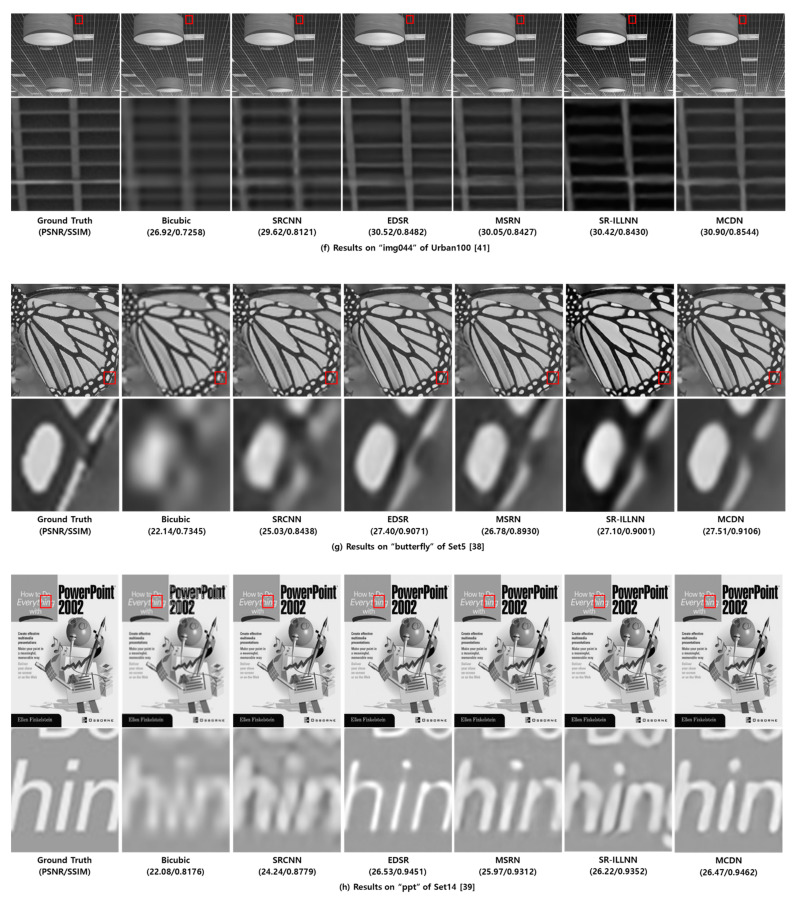

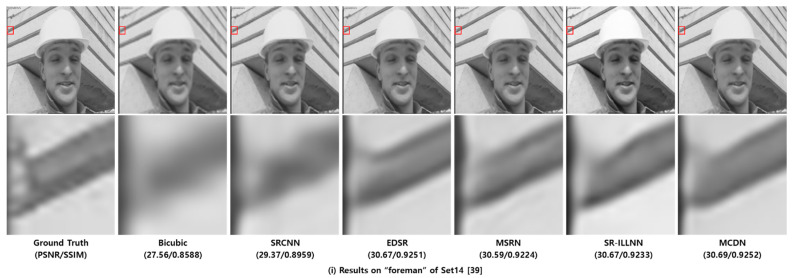
Visual comparisons on test dataset [38,39,40,41]. For each test image, the figures of the second row represent the zoom-in for the area indicated by the red box.

Figure 7 shows the examples of visual comparisons between MCDN and the previous methods including anchor on the test datasets. From the results, we verified that the proposed MCDN can recover the structural information effectively and find more accurate textures than other works.

### 4.2. Ablation Studies

In order to optimize the proposed network architectures, we conducted a variety of verification tests on the validation dataset. In this paper, we denote the number of MCDB, the number of the dense blocks per a MCDB, and the number of the dense layers per a dense block as M, D, and L, respectively. Note that the more M, D, and L are deployed in the proposed network, the more memory is required to store network parameters and the feature maps. Therefore, it is important that the optimal M, D, and L components are deployed in the proposed network to consider the trade-off between SR accuracy and network complexity.

**Figure 8:**
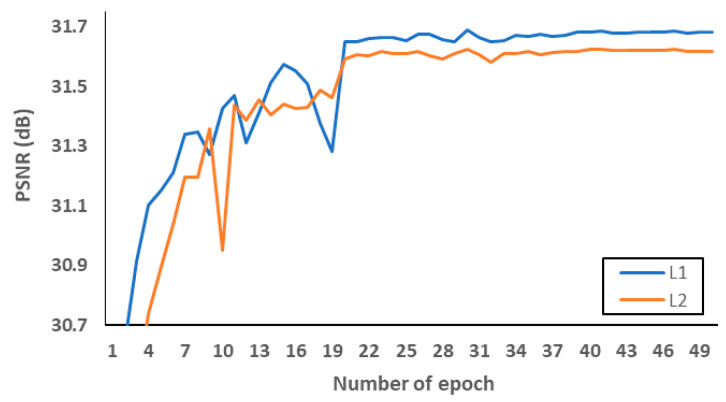
Verification of loss functions.

**Figure 9:**
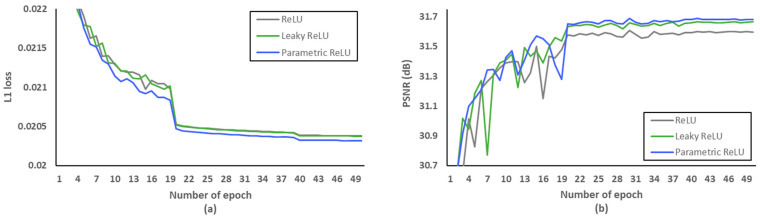
Verification of activation functions. (**a**) L1 loss per epoch. (**b**) PSNR per epoch.

Firstly, we investigated what loss functions and activation functions were beneficial to the proposed network. According to [45], L2 loss does not always guarantee better SR performance in terms of PSNR and SSIM, although it is widely used to represent PSNR at the network training stage. Therefore, we conducted PSNR comparisons to choose the well matched loss function. Figure 8 and Table 6 indicate that L1 loss can be suitable to the proposed network structure. In addition, leaky rectified linear unit (Leaky ReLU) [46] and parametric ReLU can be replaced with ReLU to avoid the gradient vanishing effect in the negative side. In order to avoid overfitting at the training stage, we evaluated L1 loss according to various epochs as shown in Figure 9a. After setting the number of epochs to 50, we measured PSNR as a SR performance in the L1 loss functions. As demonstrated in Figure 9b, we confirmed that parametric ReLU is superior to other activation functions on the proposed MCDN.

**Figure 10:**
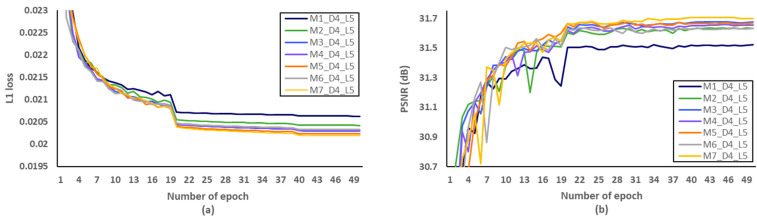
Verification of the number of MCDB (M) in terms of SR performance. (**a**) L1 loss per epoch. (**b**) PSNR per epoch.

**Figure 11:**
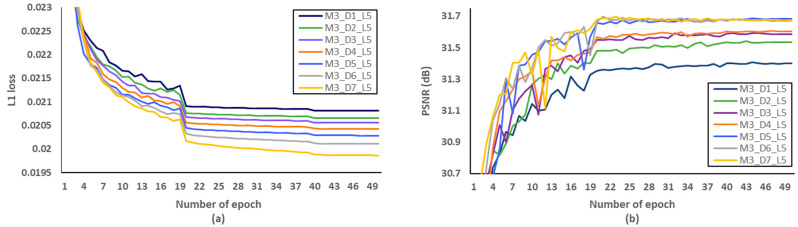
Verification of the number of dense block (D) per a MCDB in terms of SR performance. (**a**) L1 loss per epoch. (**b**) PSNR per epoch.

**Figure 12:**
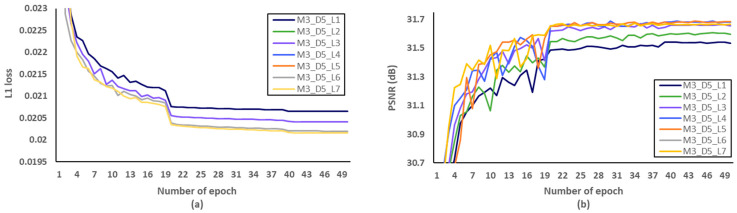
Verification of the number of dense layer (L) per a dense block in terms of SR performance. (**a**) L1 loss per epoch. (**b**) PSNR per epoch.

Secondly, we have investigated the optimal number of M, after fixing the D and L to 5 and 4, respectively. We evaluated L1 loss according to the number of epochs as shown in Figure 10a. After setting the number of epochs to 50, we measured PSNR to identify SR performance according to the various M, and Figure 10b showed that the optimal M should be set to 3. Through the evaluations of Figure 11 and Figure 12 and Table 7 and Table 8, the optimal number of D and L were set to 5 and 4 in the proposed MCDN, respectively. Consequently, the proposed MCDN can be designed to consider the trade-off between the SR performance and the network complexity as measured in Table 7, Table 8 and Table 9.

Finally, we verified the effectiveness both of skip and dense connection. The more dense connections are deployed in the between convolution layers, the more network parameters are required to compute the convolution operations. According to the results of tool-off tests on the proposed MCDN as measured in Table 10, we confirmed that both skip and dense connection have an effect on SR performance. In addition, Table 11 shows the network complexity and the inference speed according to the deployment of skip and dense connection.

## 5. Conclusions

In this paper, we proposed CNN based a multi-scale channel dense network (MCDN). The proposed MCDN aims at generating a HR image whose size is 4N × 4M given an input image N × M. It is composed of four parts, which are input layer, multi-scale channel extractor, upsampling layer, and output layer, respectively. In addition, the multi-scale channel extractor consists of three multi-scale channel dense blocks (MCDBs), where each MCDB has five dense blocks with the different channel size, and each dense block contains four dense layers. In order to design the proposed network, we extracted training images from the DIV2K dataset and investigated the trade-off between the quality enhancement and network complexity. We conducted various ablation works to find the optimal network structure. Consequently, the proposed MCDN reduced the number of parameters and the total memory size by as low as 1.2% and 17.4%, respectively while it accomplished slightly high or similar PSNR performance on most test datasets compared to EDSR. In addition, MCDN marginally reduces the total memory size by as low as 80.5% and 92.2%, respectively, compared to MSRN and SR-ILLNN with lightweight network structures. In terms of SR performances, MCDN can achieve the improved PSNR gains as high as 0.21 dB and 0.16 dB on average compared to MSRN and SR-ILLNN, respectively.

## Figures and Tables

**Table 1:** Hyper parameters of the proposed MCDN.

Hyper Parameters	Options
Loss function	L1 loss
Optimizer	Adam
Batch size	128
Num. of epochs	50
Learning rate	10^−3^ to 10^−5^
Initial weight	Xavier
Activation function	Parametric ReLU
Padding mode	Zero padding

**Table 2:** Experimental environments.

Experimental Environments	Options
Linux version	Ubuntu 16.04
Deep learning frameworks	Pytorch 1.4.0
CUDA version	10.1
Input size ($I_{\mathrm{LR}}$)	25 × 25 × 1
Label size ($I_{\mathrm{HR}}$)	100 × 100 × 1

**Table 3:** The number of parameters and total memory (MB) size.

	Num. of Parameters	Total Memory Size (MB)
SRCNN [18]	57K	14.98
EDSR [27]	43,061K	371.87
MSRN [30]	6,075K	70.56
SR-ILLNN [31]	439K	80.83
MCDN	531K	65.07

**Table 4:** Average PSNR (dB) on the test datasets. The best results of dataset are shown in bold.

Dataset	Bicubic	SRCNN [18]	EDSR [27]	MSRN [30]	SR-ILLNN [31]	MCDN
Set5	28.44	30.30	**31.68**	31.36	31.41	**31.68**
Set14	25.80	27.09	**27.96**	27.76	27.83	**27.96**
BSD100	25.99	26.86	27.42	27.36	27.33	**27.43**
Urban100	23.14	24.33	25.54	25.25	25.32	**25.56**
Average	24.73	25.80	26.70	26.49	26.54	26.70

**Table 5:** Average SSIM on the test datasets. The best results of datasets shown in bold.

Dataset	Bicubic	SRCNN [18]	EDSR [27]	MSRN [30]	SR-ILLNN [31]	MCDN
Set5	0.8112	0.8599	0.8893	0.8845	0.8848	**0.8897**
Set14	0.7033	0.7495	**0.7748**	0.7703	0.7709	0.7745
BSD100	0.6699	0.7112	**0.7309**	0.7281	0.7275	0.7305
Urban100	0.6589	0.7158	**0.7698**	0.7600	0.7583	0.7686
Average	0.6702	0.7192	0.7551	0.7489	0.7479	0.7543

**Table 6:** SR performances according to loss functions on test datasets.

	Set5		Set14		BSD100		Urban100		Average	
	PSNR	SSIM	PSNR	SSIM	PSNR	SSIM	PSNR	SSIM	PSNR	SSIM
L1	31.68	0.8897	27.96	0.7745	27.43	0.7305	25.56	0.7686	26.70	0.7543
L2	31.61	0.8883	27.90	0.7733	27.40	0.7297	25.47	0.7653	26.65	0.7524

**Table 7:** Verification of the number of dense block (D) per a MCDB in terms of network complexity.

	Num. of Parameters	Total Memory Size (MB)
M3_D1_L5	125K	25.57
M3_D2_L5	185K	34.62
M3_D3_L5	267K	44.93
M3_D4_L5	395K	57.85
M3_D5_L5	639K	76.21
M3_D6_L5	1146K	106.39
M3_D7_L5	2713K	164.02

**Table 8:** Verification of the number of dense layer (L) per a dense block in terms of network complexity.

	Num. of Parameters	Total Memory Size (MB)
M3_D5_L1	280K	37.82
M3_D5_L2	351K	45.87
M3_D5_L3	435K	54.96
M3_D5_L4	531K	65.07
M3_D5_L5	639K	76.21
M3_D5_L6	760K	88.37
M3_D5_L7	893K	101.57

**Table 9:** SR Performances on test datasets.

	Set5		Set14		BSD100		Urban100		Average	
Model	PSNR	SSIM	PSNR	SSIM	PSNR	SSIM	PSNR	SSIM	PSNR	SSIM
M1_D5_L5	31.50	0.8866	27.83	0.7714	27.34	0.7279	25.34	0.760.	26.55	0.7491
M2_D5_L5	31.58	0.8882	27.92	0.7739	27.40	0.7298	25.50	0.7665	26.66	0.7530
M3_D5_L5	31.68	0.8895	27.98	0.7747	27.43	0.7304	25.56	0.7692	26.71	0.7546
M4_D5_L5	31.66	0.8896	28.01	0.7751	27.43	0.7308	25.59	0.7708	26.73	0.7555
M5_D5_L5	31.73	0.8903	28.03	0.7755	27.44	0.7310	25.65	0.7725	26.76	0.7564
M6_D5_L5	31.70	0.8901	28.05	0.7758	27.45	0.7313	25.66	0.7729	26.77	0.7568
M7_D5_L5	31.70	0.8899	28.05	0.7761	27.44	0.7313	25.65	0.7730	26.76	0.7568
M3_D1_L5	31.40	0.8853	27.80	0.7707	27.31	0.7270	25.25	0.7576	26.50	0.7474
M3_D2_L5	31.53	0.8874	27.88	0.7724	27.36	0.7285	25.36	0.7616	26.58	0.7500
M3_D3_L5	31.58	0.8878	27.90	0.7731	27.39	0.7292	25.41	0.7638	26.61	0.7514
M3_D4_L5	31.60	0.8883	27.96	0.7742	27.40	0.7299	25.50	0.7665	26.66	0.7531
M3_D5_L5	31.68	0.8895	27.98	0.7747	27.43	0.7304	25.56	0.7692	26.71	0.7546
M3_D6_L5	31.67	0.8894	27.99	0.7749	27.43	0.7308	25.59	0.7708	26.72	0.7555
M3_D7_L5	31.67	0.8897	27.95	0.7748	27.41	0.7307	25.58	0.7711	26.71	0.7556
M3_D5_L1	31.53	0.8871	27.86	0.7722	27.35	0.7283	25.37	0.7615	26.58	0.7499
M3_D5_L2	31.59	0.8880	27.90	0.7732	27.38	0.7292	25.43	0.7642	26.62	0.7516
M3_D5_L3	31.65	0.8891	27.93	0.7739	27.41	0.7299	25.50	0.7667	26.67	0.7531
M3_D5_L4	31.68	0.8897	27.96	0.7745	27.43	0.7305	25.56	0.7686	26.70	0.7543
M3_D5_L5	31.68	0.8895	27.98	0.7747	27.43	0.7304	25.56	0.7692	26.71	0.7546
M3_D5_L6	31.68	0.8897	27.99	0.7750	27.43	0.7309	25.60	0.7706	26.73	0.7555
M3_D5_L7	31.66	0.8894	27.99	0.7753	27.43	0.7309	25.61	0.7711	26.73	0.7557

**Table 10:** SR performances according to tool-off tests.

Skip Connection	Dense Connection	Set5		Set14		BSD100		Urban100		Average	
		PSNR	SSIM	PSNR	SSIM	PSNR	SSIM	PSNR	SSIM	PSNR	SSIM
Disable	Disable	26.42	0.7362	24.34	0.6297	24.78	0.5985	21.95	0.5823	23.50	0.5963
Disable	Enable	31.37	0.8845	27.78	0.7698	27.29	0.7264	25.22	0.7557	26.47	0.7462
Enable	Disable	31.59	0.8879	27.90	0.7731	27.39	0.7291	25.42	0.7643	26.62	0.7516
Enable	Enable	31.68	0.8897	27.96	0.7745	27.43	0.7305	25.56	0.7686	26.70	0.7543

**Table 11:** Network complexity and inference speed on BSD100 according to tool-off tests.

Skip Connection	Dense Connection	Num. of Parameters	Total Memory Size (MB)	Inference Speed (s)
Disable	Disable	167K	40.02	24.09
Disable	Enable	531K	65.07	46.59
Enable	Disable	434K	40.02	26.37
Enable	Enable	531K	65.07	47.20

## Data Availability

Not applicable.
